# Fine and gray competing risk regression model of predictors of loss to follow up among female sex workers receiving antiretroviral therapy in Nigeria: 2016–2022

**DOI:** 10.1186/s12905-026-04432-z

**Published:** 2026-04-02

**Authors:** Wingston Felix Ng’ambi, Abiye Kalaiwo, Janne Estill, Erol Orel, Chigere Adoration, Kene David Nwosu, Olivia Keiser

**Affiliations:** 1 Institute of Global Health, University of Geneva, Geneva, Switzerland; 2 Global Research and Analyses for Public Health (GRAPH) Network, University of Geneva, Geneva, Switzerland; 3 Heartland Alliance LTD/GTE (HALG), Abuja, Nigeria; 4 Department of Health Systems and Policy, Health Economics and Policy Unit, Kamuzu University of Health Sciences, Lilongwe, Malawi

**Keywords:** Female sex workers, ART outcomes, Nigeria, Sub-Saharan Africa, Loss-to-follow-up

## Abstract

**Introduction:**

Globally loss to follow up (LTFU) in HIV treatment is a profound challenge in the era of expanded HIV treatment coverage amongst different populations including female sex workers (FSWs). Therefore, we conducted this study to assess the predictors of LTFU among female sex workers on antiretroviral therapy in Nigeria between 1 January 2016 and 31 August 2022.

**Methods:**

A retrospective cohort analysis of routine HIV program data evaluating the HIV treatment outcomes of FSWs from Bayelsa, Akwa Ibom, Cross Rivers, Niger and Lagos states in Nigeria. We fitted multivariable competing risk regression analyses for the rate of LTFU using death, stopping ART, and transferring to another clinic as competing events.

**Results:**

A total of 16,727 FSWs were included in this analysis. By 31 August 2022, 15,655 (94%) were alive and under follow-up, 230 (1%) were lost to follow-up (LTFU), 87 (< 1%) had died, 481 (3%) had transferred to other clinics for ART, and 274 (2%) had stopped ART. The median follow-up time was 20 months (IQR: 13–27). Compared with FSWs from other states, those from Niger State had a lower risk of LTFU (adjusted subdistribution hazard ratio [aSHR] 0.56; 95% CI: 0.49–0.64). FSWs aged ≥ 30 years had a higher risk of LTFU than those aged 15–29 years (aSHR 1.24; 95% CI: 1.16–1.33). Migrants had a lower risk of LTFU than non-migrants (aSHR 0.69; 95% CI: 0.64–0.74). Compared with FSWs with no formal education, those with primary, secondary, and tertiary education had lower risks of LTFU, with adjusted SHRs of 0.21 (95% CI: 0.19–0.24), 0.42 (95% CI: 0.38–0.46), and 0.54 (95% CI: 0.48–0.60), respectively. FSWs with hypertension had a higher risk of LTFU (aSHR 1.27; 95% CI: 1.20–1.35), whereas those with normal BMI had a lower risk than underweight FSWs (aSHR 0.70; 95% CI: 0.65–0.74).

**Conclusion:**

Retention on ART was 94%, with 15,655 of 16,727 FSWs remaining alive and under follow-up by 31 August 2022, although risk of LTFU varied across subgroups. Higher risk of LTFU was observed among FSWs aged ≥ 30 years, students, and those with hypertension, while lower risk was observed among FSWs from Niger State, migrants, unemployed FSWs, and those with normal BMI. Programs in similar settings may consider adapting implementation features of the Nigerian FSW program while strengthening support for subgroups at higher risk of LTFU.

## Introduction

Female sex workers (FSWs) bear a disproportionately large burden of HIV infection globally [1, 2]. The underlying causes of this heavy HIV burden are multifaceted, spanning behavioural, social, and structural realities that affect sex workers of all genders. According to a 2018 systematic review and meta-analysis, the global HIV prevalence among cisgender women who work as sex workers is 10.4% (95% CI: 9.5–11.5). The highest regional HIV prevalence is observed in Eastern and Southern Africa (33.3%; 95% CI: 29.2–37.6), followed by West and Central Africa (20.1%; 95% CI: 16.7–23.8) [3]. In recent years the coverage of HIV treatment amongst the various populations including FSWs in Africa has been increasing [4]. Furthermore, most countries of Africa have implemented the HIV test-and-treat policy to use treatment as prevention of further spread of HIV as well as expanding the ART coverage [5, 6].

FSWs are a known high-risk group who are at increased risk of HIV acquisition due to exposure to multiple sexual partners and inability to negotiate safe sex attributed to challenging economic circumstances [7, 8]. Settings like Tanzania and Malawi have reported increased uptake and linkage to HIV services among FSWs following the 2016 WHO consolidated guidelines for HIV [9]. In Sub-Saharan Africa, FSWs have the highest HIV prevalence among the main high-risk groups with almost 40% of the FSWs living with the virus [8]. In West Africa, 10–32% of new HIV infections have been estimated to result from sex work [10]. In Nigeria, the Integrated Biological and Behavioral Surveillance Survey reported a higher prevalence of HIV among brothel-based female sex workers (19.4%) than among non-brothel-based female sex workers (8.6%) [11]. Although providing ART for HIV-infected FSWs is important both as a HIV prevention strategy and to treat the infected FSWs themselves, the FSWs face a myriad of challenges that impede on their retention in ART care [12]. Some of these hindrances to the overall HIV care cascade include stigma, migration, criminalization, violence, and substance use, which present increasingly concurrent risks with HIV among sex workers [13]. The steps in the HIV treatment cascade are HIV testing, diagnosis, referral to HIV health services, start of HIV antiretroviral therapy (ART), and continuation of HIV treatment to maintain viral suppression [14]. Within the HIV care cascade, several HIV programmes have found it relatively easy to test FSWs for HIV and enroll those infected on ART [2], however, retaining FSW who started ART in care has been one of the greatest hurdles of the HIV continuum of care. Some of the factors that have been reported to increase the likelihood of FSWs to disengage from ART are mobility, irregular working hours, and difficult or completely absent family relationships [15]. According to pooled estimates, roughly 38% (95% CI: 29–48) of HIV-infected sex workers were receiving treatment at the time, roughly 76% (95% CI: 68–83) of those receiving treatment were adhering, and roughly 57% (95% CI: 46–68) of those adhering were virally suppressed [16].

Nigeria has the second largest HIV epidemic globally with about 1.9 million persons of all ages living with the virus by the end of 2021 [17]. Adult HIV prevalence in Nigeria is nevertheless much lower (1.3%) than in other sub-Saharan African countries such as South Africa (19%) and Zambia (11.5%) [18]. Recent drops in HIV prevalence estimates in Nigeria have been attributed to better long-term HIV/AIDS surveillance [18] and the availability of antiretroviral medications for the management of HIV across the globe since 2002 [19]. Although studies have assessed HIV prevalence amongst FSWs [20, 21], ART treatment outcomes have not been fully investigated in Nigeria or other similar settings [22–24]. A systematic review that assessed ART uptake, attrition, adherence and outcomes among FSWs living with HIV suggested that there is a concerning lack of published data for treatment response for FSWs [8, 20, 25]. Furthermore, although important progress has been made in biomedical interventions, limited data on ART retention suggest that sustained investment in community and structural interventions aimed at ensuring high ART retention may benefit FSWs and other key populations.

A key knowledge gap is that predictors of LTFU among FSWs on ART in Nigeria have not been examined using methods that account for competing ART outcomes. In this setting, FSWs may die, stop ART, or transfer to another clinic before becoming LTFU, and these events prevent LTFU from occurring as the first observed outcome. Traditional survival models typically censor such events and may therefore misestimate the risk of LTFU [26]. Competing-risk analysis provides additional insight by modelling the cumulative incidence of LTFU while explicitly accounting for these alternative outcomes [27]. This approach is especially relevant for FSWs, whose care trajectories are often shaped by mobility and treatment interruptions. Therefore, we conducted this study to assess predictors of LTFU among FSWs receiving ART in Nigeria between 1 January 2016 and 31 August 2022 using a Fine and Gray competing-risk regression model.

## Methods

### Study design

We conducted a retrospective cohort study using routine programme data to identify factors linked to loss to follow up among female sex workers starting antiretroviral therapy in Nigeria between 1 January 2016 and 31 August 2022.

### Study setting

**Fig. 1:**
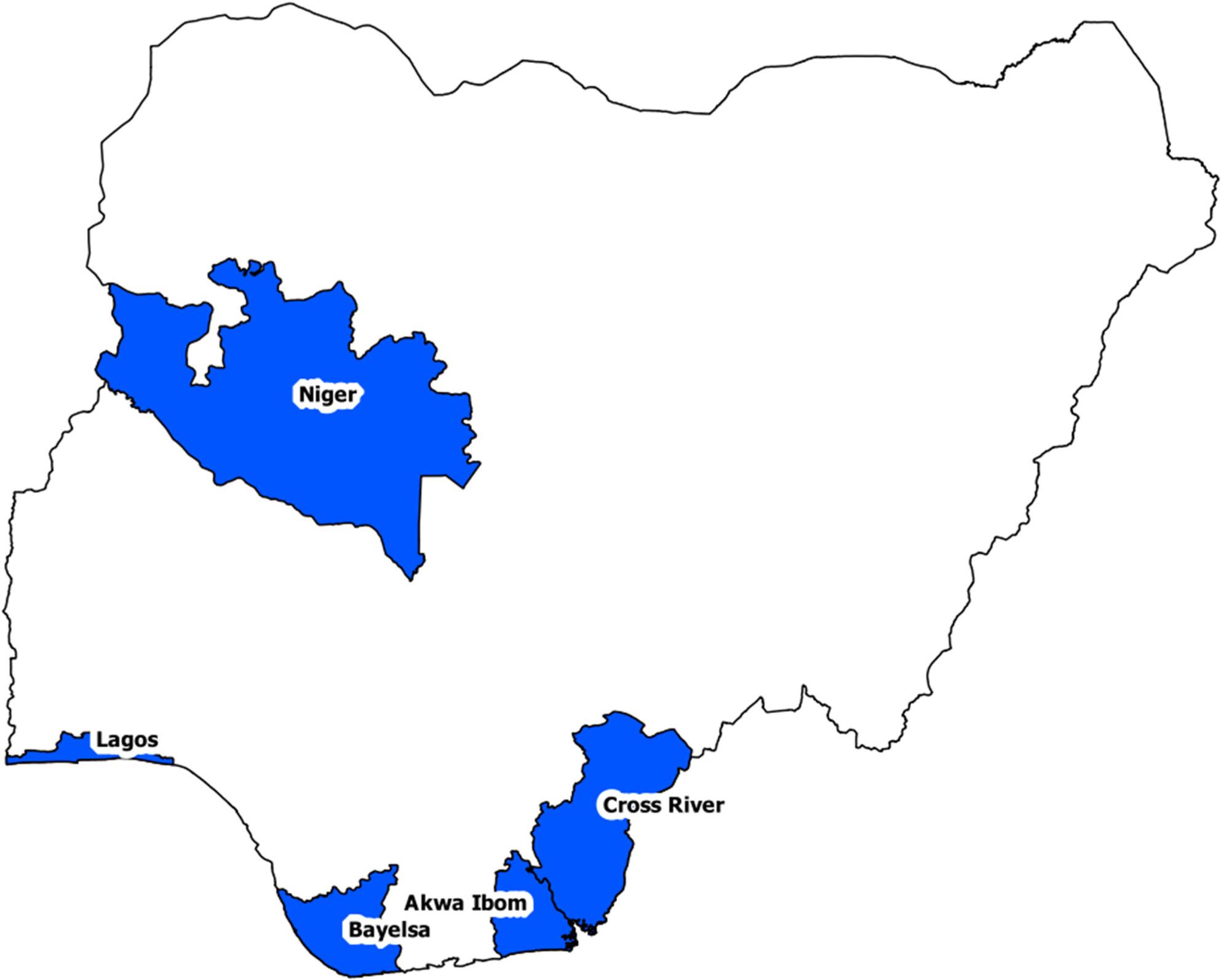
States in which the key population program is implemented in Nigeria in 2022

This study that evaluates the HIV treatment outcomes of FSWs in five of Nigeria’s 36 states (Fig. 1): Bayelsa, Akwa Ibom and Cross Rivers in the South-South region; Niger in the North-Western region; and Lagos state is in the South-West region of Nigeria. There is no national key populations program in the North-East and South-East regions due to security challenges and lower HIV prevalence. Therefore, the United States President’s Emergency Plan for AIDS Relief (PEPFAR) prioritized the implementation of Nigeria’s key population (KP) program in the five states mentioned above.

### HIV/AIDS management amongst FSWs in Nigeria

Since 2010, the Nigerian government has been implementing the Minimum Prevention Package Intervention (MPPI) in response to the HIV pandemic [28]. The MPPI uses behavioral, biomedical, and structural interventions in addressing HIV prevention. The provision of the HIV/AIDS services to the FSWs is through clinics owned by the public or community-based organizations (CBOs) [29]. The CBOs use peer FSWs in their programmes and can therefore reach the FSWs easier than other providers. Furthermore, Nigeria follows the national and international guidelines in the treatment and care of persons living with HIV/AIDS regardless of whether they belong to KPs or not [30]. Nigeria has also committed to achieve the UNAIDS 95–95–95 goal – of having 95% of people living with HIV diagnosed, 95% of those diagnosed on antiretroviral therapy (ART), and 95% of those treated virally suppressed by the year 2030 [31]. Some other interventions targeting FSWs that have been implemented include pre-exposure prophylaxis (PrEP) and HIV self-testing (HIVST). PrEP has been available for FSWs in Nigeria since 2017 and HIVST since 2018 [30].

### Data collection

The National Agency for the Control of AIDS (NACA) in Nigeria oversees the collection of data pivotal to monitor and evaluate Nigeria’s HIV/AIDS programs. In Nigeria, the Heartland Alliance focuses on collecting HIV data from key populations (KPs), who are at increased risk of HIV infection. The key populations targeted by the Heartland Alliance include men who have sex with men (MSM); FSWs; people who inject drugs (PWID); transgender individuals; and individuals in prisons and other closed settings. The data for the KPs are captured electronically using the electronic medical records from the states included in this study.

### Data management

We included all FSWs who were enrolled in the ART programme and followed up between 1 January 2016 and 31 August 2022 from the Nigerian states shown in Fig. 1. The following variables were collected at baseline: age, state, highest level of education (none; primary; secondary; tertiary), internal migration status (having migrated to the current place of residence or not), year of ART initiation, WHO HIV clinical stage (1, 2, 3, 4), occupation (student; employed; unemployed), body mass index (BMI; underweight < 18.4; normal 18.4–23.9; overweight 24 + kg/m^2^), hypertension (normal or high blood pressure); HIV viral suppression (suppressed i.e. less than 1000 or unsuppressed i.e. at least 1000 copies per ml of blood), marital status (single; married). The blood pressure was considered to be normal if the systolic blood pressure was < 120 or diastolic blood pressure was < 80, and high otherwise. We categorized age into two age groups (15–29 and 30 + years). The outcome variable for this analysis was the antiretroviral outcome (alive or transfer to another clinic, lost to follow-up (LTFU), died, or stopped treatment). For Fine and Gray regression modeling, the outcome of interest is *LTFU* while *died*,* stopped ART and transfer to another clinic* are competing events. The predictor variables were age, WHO HIV clinical stage, state (Fig. 1), internal migration status, marital status, highest level of education, occupation, and year of ART initiation. For the analysis, we considered to combine Bayelsa, Akwa Ibom, Cross Rivers and Lagos states located next to each other if the number of LTFU cases in the individual states was low (i.e. dichotomize the variable into Niger vs. others).

### Data analysis

The data were managed and analysed in R software v4.2 [32]. A descriptive analysis [33] was first performed detailing the characteristics of the FSWs. Multiple imputation chained equations, with 20 imputation rounds and 5000 permutations, were used to impute missing data of the following co-variables: occupation, education level, baseline BMI category, and baseline HIV viral suppression status. Time-to-event was defined as the interval from ART initiation to the first recorded episode of LTFU. Participants were followed from the date of ART initiation until LTFU, the occurrence of a competing event (death, stopping ART, or transfer to another clinic), or administrative censoring on 31 August 2022 if LTFU had not occurred. Fine and Gray competing-risk regression models were used to estimate sub-distribution hazard ratios for LTFU while accounting for competing events. The covariates considered in the multivariable analysis were state of residence, HIV viral suppression, age group, highest education level, migration status, occupation, year of ART initiation, marital status, WHO HIV clinical stage, BMI category, and hypertension category. Covariates were initially selected a priori based on prior literature and similar models [27, 34]. We then fitted a full multivariable Fine and Gray model and used backward selection with Wald tests to derive the final model across multiply imputed datasets.

We first fitted a bivariate analysis of each of the independent variables and competing risk for the incidence of LTFU in the programme using the Fine & Gray (FG) regression model [35]. The variables for the final model were chosen using a stepwise procedure. First, we fitted a complete multivariable model to find the p-values for each predictor variable. In this analysis, we reported sub hazard ratios, written as SHR. An SHR shows how a predictor changes the rate of a specific event while taking competing risks into account. It is interpreted in the same simple way as a hazard ratio, but it adjusts for the fact that some participants may experience other events that prevent the outcome of interest.

We then used the Wald test to compare layered models and implement backward variable selection. Excluding each variable from the whole model and evaluating the difference in model fit allowed for the calculation of its p-value. In contrast to the smaller, nested model, the Wald test offered a statistical comparison to assess if the extra predictors in the bigger model substantially improved the fit. The Wald test was used to compare nested models in the context of multiply imputed datasets [36]. Statistical significance was set at *P* < 0.05.

### Ethical consideration

This secondary analysis was based on routinely collected programmatic data from Nigeria’s national Key Population program. Only de-identified data were accessed by the study team to ensure confidentiality. Ethical approval for the study was obtained from the Federal Capital Territory Health Research Ethics Committee, Nigeria (Approval No: FHREC/2023/01/127/20-07-23).

## Results

### Baseline characteristics of female sex workers who received antiretroviral therapy in Nigeria between 2016 and 2022

The baseline characteristics of FSWs receiving ART in Nigeria between 2016 and 2022 are shown in Table 1. A total of 16,727 FSWs received ART during this period. Of these, 11.9% were from Niger State and 88.1% were from other states. Overall, 30.4% were aged 15–29 years. At enrolment, 78.7% had unsuppressed viral loads. Most participants were single (88.0%), while 11.7% were married. Regarding education, 6.7% had no formal education and 55.4% had completed secondary education. In addition, 67.5% had migrated to their current state of residence, while 32.5% had not. Students accounted for 6.5% of the cohort, and 52.7% were employed. The majority, initiated ART in 2020 (50.9%). Almost all participants were classified as WHO HIV clinical stage 1 (96.1%), while 0.3% were in stage 3; no participants were in stage 4. At enrolment, 4.5% were underweight and 48.7% had normal BMI. The proportions with normal blood pressure (50.5%) and high blood pressure (49.5%) were similar. The median age was 33 years (interquartile range [IQR]: 28–39). Missing information ranged from 0.4% to 9.5% across variables.

**Table 1:** Characteristics of female sex workers who started antiretroviral therapy in Nigeria between 01 January 2016 and 31 August 2022

Characteristics	n	Percent
Total	16727	100.0
State of residence		
*Other states*	14737	88.0
*Niger*	1990	12.0
Age group		
* 15-29*	5088	30.0
* 30+*	11639	70.0
Marital status		
* Single*	14715	88.0
* Married*	1953	11.6
* Missing*	59	0.4
Highest education level		
* None*	1126	6.7
* Primary*	1871	11.2
* Secondary*	9263	55.4
* Tertiary*	3779	22.6
* Missing*	688	4.1
Migrated to current state of residence		
* No*	5432	32.0
* Yes*	11295	68.0
Occupation		
* Employed*	8817	52.7
* Unemployed*	6586	39.4
* Student*	1082	6.5
* Missing*	242	1.4
Year of ART initiation		
* 2016*	472	2.8
* 2017*	1832	11.0
* 2018*	895	5.4
* 2019*	3531	21.1
* 2020*	8509	50.9
* 2021*	1412	8.4
* 2022*	76	0.5
WHO HIV clinical stage		
* Stage 1*	16080	96.1
* Stage 2*	604	3.6
* Stage 3*	43	0.3
BMI categories (kg/m^2^) at enrolment		
* Underweight (<18.4)*	758	4.5
* Normal (18.4-23.9)*	8150	48.7
* Overweight (24.0+)*	6328	37.8
* Missing*	1491	8.9
Hypertension categories at enrolment		
* Normal (*systolic: <120 or diastolic: <80)	8455	50.5
* High (*systolic: ≥120 or diastolic: ≥80*)*	8272	49.5
HIV viral load suppression at enrolment		
* Suppressed *	1963	11.7
* Unsuppressed *	13168	78.7
* Missing*	1596	9.5

*BMI *Body mass index, *WHO *World Health Organisation, *HIV *Human Immunodeficiency Virus, *ART *Antiretroviral therapy

### Primary antiretroviral treatment outcomes for female sex workers

The median follow-up time was 20 months (IQR: 13–27). Of the 16,727 FSW initiated on ART; 15,655 (94%) were alive and under follow-up, 230 (1%) were LTFU, 87 (< 1%) died, 481 (3%) moved to access ART from other clinics, and 274 (2%) stopped ART by 31 August 2022. The distribution of cumulative ART outcomes across socio-economic characteristics of the FSWs is shown in Table 2. Those who were older, had less education, were unemployed, started ART earlier, and had a higher WHO HIV stage were more likely to experience competing outcomes, namely loss to follow-up (LTFU), death, or quitting ART. Moreover, LTFU, mortality, and ART cessation rates were somewhat higher among singles, migrants, and people with high blood pressure.

**Table 2:** Outcomes of antiretroviral therapy for the female sex workers in Nigeria using complete case analysis: 01 January 2016 and 31 August 2022

Characteristics (n=30,125)	Aliven (%)	LTFU n (%)	Dead n (%)	Stoppedn (%)
State of residence				
Other states	27,805 (98.9)	166 (0.6)	52 (0.2)	83 (0.3)
Niger	1,990 (98.6)	29 (1.4)	0 (0.0)	0 (0.0)
HIV viral suppression at enrolment				
Suppressed	3,446 (97.1)	37 (1.0)	25 (0.7)	40 (1.1)
Unsuppressed	26,349 (99.1)	158 (0.6)	27 (0.1)	43 (0.2)
Age group				
0-29	9,112 (99.0)	55 (0.6)	16 (0.2)	24 (0.3)
30+	20,683 (98.9)	140 (0.7)	36 (0.2)	59 (0.3)
Highest education level				
None	1,536 (97.9)	20 (1.3)	2 (0.1)	11 (0.7)
Primary	3,379 (98.9)	22 (0.6)	5 (0.1)	9 (0.3)
Secondary	17,948 (99.1)	106 (0.6)	26 (0.1)	37 (0.2)
Tertiary	6,932 (98.7)	47 (0.7)	19 (0.3)	26 (0.4)
Migrated to current state of residence				
No	9,875 (99.0)	48 (0.5)	18 (0.2)	29 (0.3)
Yes	19,920 (98.8)	147 (0.7)	34 (0.2)	54 (0.3)
Occupation				
Employed	14,766 (98.9)	109 (0.7)	27 (0.2)	28 (0.2)
Unemployed	13,289 (98.9)	80 (0.6)	23 (0.2)	50 (0.4)
Student	1,740 (99.3)	6 (0.3)	2 (0.1)	5 (0.3)
Year of ART initiation				
2016	485 (92.7)	13 (2.5)	7 (1.3)	18 (3.4)
2017	2,089 (97.2)	28 (1.3)	12 (0.6)	20 (0.9)
2018	1,266 (97.6)	12 (0.9)	7 (0.5)	12 (0.9)
2019	5,425 (99.0)	24 (0.4)	14 (0.3)	18 (0.3)
2020	12,349 (99.3)	71 (0.6)	7 (0.1)	10 (0.1)
2021	6,382 (99.2)	39 (0.6)	5 (0.1)	5 (0.1)
2022	1,799 (99.6)	8 (0.4)	0 (0.0)	0 (0.0)
Marital status				
Single	26,332 (98.9)	168 (0.6)	42 (0.2)	74 (0.3)
Married	3,463 (98.7)	27 (0.8)	10 (0.3)	9 (0.3)
WHO HIV clinical stage				
Stage 1	28,829 (99.0)	181 (0.6)	41 (0.1)	69 (0.2)
Stage 2	913 (96.3)	13 (1.4)	8 (0.8)	14 (1.5)
Stage 3	53 (93.0)	1 (1.8)	3 (5.3)	0 (0.0)
BMI categories at enrolment				
Underweight (<18.4)	1,225 (97.7)	11 (0.9)	12 (1.0)	6 (0.5)
Normal (18.4-23.9)	16,443 (98.8)	123 (0.7)	27 (0.2)	50 (0.3)
Overweight (24.0+)	12,127 (99.2)	61 (0.5)	13 (0.1)	27 (0.2)
Hypertension categories at enrolment				
Normal (systolic: <120 or diastolic: <80)	14,904 (99.0)	92 (0.6)	24 (0.2)	38 (0.3)
High (systolic: ≥120 or diastolic: ≥80)	14,891 (98.8)	103 (0.7)	28 (0.2)	45 (0.3)

*BMI *Body mass index, *WHO *World Health Organisation, *HIV *Human Immunodeficiency Virus, *ART *Antiretroviral therapy, *LTFU *Loss to follow-up

### Cumulative incidence functions of competing events

**Fig. 2:**
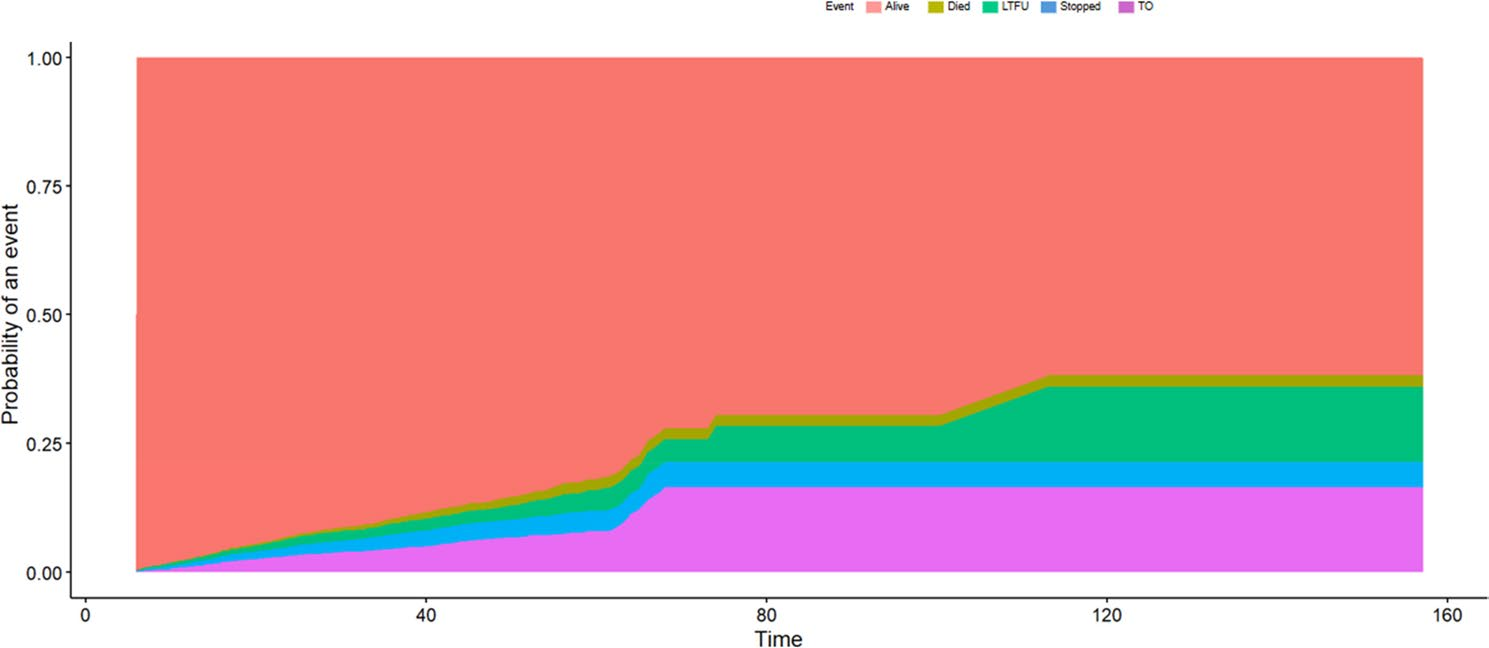
Cumulative incidence functions of competing risk events for the Nigeria female sex workers: 2016–2022. Events included: Alive or retained in care; LTFU – loss to follow-up; Died – mortality; Stopped – discontinued ART; TO – transferred out

The cumulative incidence functions (CIF) of competing events are shown in Fig. 2. According to the CIF analysis, most participants in the cohort stay in the initial state (i.e. being on ART) over time, but the likelihood of competing outcomes; death, LTFU, ART cessation, and TO; increases steadily over time, with a notable increase in LTFU and ART discontinuation between 40 and 80 time units, indicating critical attrition periods; mortality is a persistent but relatively lower-risk outcome in comparison to other competing events; a sizable percentage of patients transfer out to other facilities, which might be due to movement within the healthcare system rather than treatment failure.

### Factors associated with competing risk of being LTFU

The factors associated with loss to follow-up amongst the FSW in Nigeria who started ART between 2016 and 2022 are shown in Table 3. State of residence, age, education level, migration to current state of residence, occupation, marital status, hypertension category, year of ART initiation, WHO HIV clinical stage, and body mass index were the factors associated with the likelihood of being lost to follow-up in the multivariable model. FSWs from Niger state were less likely to be LTFU compared to the FSWs from the other states (aSHR = 0.56, 95%CI: 0.49–0.64). On the other hand, the FSWs who were aged at least 30 years were more often LTFU compared to those younger (aSHR = 1.24, 95%CI: 1.16–1.33). Compared with FSWs with no formal education, those with primary, secondary, and tertiary education had lower risks of LTFU (Table 3). The FSWs who migrated to another state of residence were less likely to be LTFU than those living in their original state of residence (aSHR = 0.69, 95%CI: 0.64–0.74). Students were more likely (aSHR = 1.46, 95%CI: 1.37–1.56) and FSWs who were unemployed were less likely (aSHR = 0.65, 95%CI: 0.55–0.76,) to be LTFU compared to the FSWs that were employed.

**Table 3:** Univariable and multivariable competing risk regression analysis for predictors of loss to follow-up among HIV-positive female sex workers in Nigeria between 01 January 2016 and 31 August 2022

Characteristics (n=16,727)	Crude		Adjusted	
	SHR (95%CI)	P-value	SHR (95%CI)	P-value
State of residence				
* Other states*	1.00		1.00	
* Niger*	0.75 (0.69-0.82)	<0.0001	0.56 (0.49-0.64)	<0.0001
HIV viral suppression				
* Suppressed*	1.00			
* Unsuppressed*	0.03 (0.46-0.53)	<0.0001		
Age group				
* 0-29*	1.00		1.00	
* 30+*	1.39 (1.29 -1.48)	<0.0001	1.24 (1.16-1.33)	<0.0001
Highest education level				
* None*	1.00		1.00	
* Primary*	0.26 (0.23-0.29)	<0.0001	0.21 (0.19-0.24)	<0.0001
* Secondary*	0.39 (0.36-0.42)	<0.0001	0.42 (0.38-0.46)	<0.0001
* Tertiary*	0.44 (0.40-0.48)	<0.0001	0.54 (0.48-0.60)	<0.0001
Migrated to current state of residence				
* No*	1.00	<0.0001	1.00	
* Yes*	0.67 (0.62-0.71)		0.69 (0.64-0.74)	<0.0001
Occupation				
* Employed*	1.00		1.00	
* Student*	1.67(1.57-1.78)	<0.0001	1.46 (1.37-1.56)	<0.0001
* Unemployed*	0.61(0.52-0.72)	<0.0001	0.65 (0.55-0.76)	<0.0001
Year of ART initiation				
* 2016*	1.00		1.00	
* 2017*	1.24 (1.12-1.37)	<0.0001	1.25 (1.13-1.39)	<0.0001
* 2018*	0.39 (0.33-0.45)	<0.0001	0.46 (0.40-0.54)	<0.0001
* 2019*	0.15 (0.13 0.17)	<0.0001	0.16 (0.14-0.19)	<0.0001
* 2020*	0.37 (0.33-0.42)	<0.0001	0.32 (0.29-0.37)	<0.0001
* 2021*	0.45 (0.38-0.54)	<0.0001	0.43 (0.34-0.50)	<0.0001
* 2022*	1.05 (0.78 1.42)	0.750	1.69 (1.26-2.28)	0.004
Marital status				
* Single*	1.00		1.00	
* Married*	1.21 (1.12-1.31)	<0.0001	1.16 (1.07-1.26)	<0.0001
WHO HIV clinical stage				
* Stage 1*	1.00		1.00	
* Stage 2*	1.32 (1.19-1.48)	<0.0001	0.88 (0.78-0.98)	0.029
* Stage 3*	2.68 (2.08-3.47)	<0.0001	0.90 (0.69-1.15)	0.40
BMI categories (kg/m^2^)				
* Underweight (<18.4)*	1.00		1.00	
* Normal (18.4-23.9)*	0.69 (0.65-0.73)	<0.0001	0.70 (0.65-0.74)	<0.0001
* Overweight (24.0+)*	1.18 (1.06-1.31)	0.003	0.98 (0.87-1.10)	0.67
Hypertension categories				
* Normal (*systolic: <120 or diastolic: <80)	1.00		1.00	
* High (*systolic: ≥120 or diastolic: ≥80*)*	1.28 (1.21-1.36)	<0.0001	1.27 (1.20-1.35)	<0.0001

*SHR *Sub-Hazard Ratio, *BMI *Body mass index, *WHO *World Health Organisation, *HIV *Human Immunodeficiency Virus, *ART *Antiretroviral therapy, *95%CI *95 per cent confidence interval

The likelihood of LTFU was highest among FSWs who were enrolled in either 2017 or 2022, and lowest among those who started ART between 2018 and 2021 (Table 3). Married FSWs were more likely to be lost to follow-up than single FSWs (aSHR 1.16; 95% CI: 1.07–1.26). FSWs who were in WHO HIV clinical stage 2 were less likely to be lost to follow-up compared to those who were in clinical stage 1 (aSHR = 0.88, 95%CI: 0.78–0.98) while among FSWs in clinical stage 3 the likelihood of loss to follow-up was similar to that of those in clinical stage 1 (aSHR = 0.90, 95%CI: 0.69–1.15). The FSWs with normal BMI were less likely to be lost to follow-up than those who were underweight (aSHR = 0.70, 95%CI: 0.65–0.74) while those with overweight had no difference in LTFU compared with those underweight (aSHR = 0.98, 95%CI: 0.87–1.10). The FSWs who had high blood pressure were more likely to be lost to follow-up than those with normal blood pressure (aSHR = 1.27, 95%CI: 1.20–1.35).

## Discussion

This study analyzed the incidence and predictors of LTFU in the presence of other competing outcomes (i.e. death, stopping ART and transferring to another clinic) in the FSW ART program in Nigeria. The following were the key findings of the study: retention on ART was very high; FSWs from Niger State and those with higher levels of education were less likely to be LTFU; older FSWs (≥ 30 years), students, and those with high blood pressure were at higher risk of LTFU compared to their counterparts; FSWs with a history of internal migration to their current place of residence were less likely to be LTFU than non-migrants; FSWs with normal BMI had a lower risk of LTFU compared to those underweight; and FSWs in WHO HIV clinical stage 2 were less likely to be LTFU compared to stage 1, while no significant difference was observed for stage 3. Competing outcomes were more common among older, less educated, unemployed individuals, early ART initiators, those with advanced HIV, singles, migrants, and those with high blood pressure. There are policy and practical implications of the findings as discussed below.

Like in many other countries in SSA, Nigeria experiences high rates of death, ART cessation, and LTFU among FSWs due to structural and health-related impediments to sustained HIV treatment participation [37]. Due to comorbidities, treatment exhaustion, or extended HIV infection, older FSWs frequently have higher mortality rates and ART dropout rates [38]. Because health literacy and financial stability are essential for obtaining care, low levels of education may impede ART adherence, while the observed lower risk of LTFU among unemployed FSWs in our cohort may reflect context-specific differences in care access, availability for follow-up, or program engagement [39]. Despite being helpful in lowering long-term morbidity, early ART initiation is linked to LTFU in key populations because of initial stigma, adverse effects, and insufficient follow-up procedures [40]. According to Belay and Derebe (2022), higher WHO HIV stages upon enrolment signify significant disease development, which raises the probability of death and treatment cessation [41]. Furthermore, adherence and LTFU may be worse among migrants and singles due to their frequently inferior social support systems [42]. Due to mobility and stigma in new places, migration, which is widespread among FSWs, interferes with continuity of care [42]. By raising mortality risks and treatment complexity, hypertension, a non-communicable condition that is on the rise in SSA, makes managing HIV even more difficult [43, 44]. Targeted interventions, such as economic empowerment initiatives, differentiated care models, and mobile health tactics catered to the particular requirements of FSWs in Nigeria and throughout SSA, are necessary to address these inequities.

The effectiveness of focused interventions in this vulnerable group is demonstrated by our study’s noteworthy 94% retention rate in ART care among FSWs in Nigeria’s Key Population Programme. Given the challenges of meeting healthcare requirements in marginalised populations—such as stigma, mobility, and socioeconomic disparities—this result is especially remarkable. This could be due to the utilization of community-based ART interventions or the use of FSWs as peer educators in ART programmes as previously reported in SSA by Atuhaire et al. [7], Lancaster et al. [22] and Ibiloye et al. [23]. The results are in line with research conducted throughout SSA, where targeted ART initiatives have continuously shown higher retention rates among important populations. Studies conducted in South Africa and Kenya, for example, show comparable results when healthcare models are modified to suit the particular challenges faced by FSWs, such as mobile outreach programs, peer support networks, and flexible clinic hours [45–47]. Further supporting regional evidence that socioeconomic empowerment improves health outcomes in important groups, our study highlights the beneficial effects of migration and higher education levels on ART retention [48]. These results highlight how important inclusive, customised healthcare strategies are to meeting UNAIDS’ 95-95-95 goals and improving equity in the provision of HIV care. In our current study, the HIV programme for the FSW included peer support, making it easier to track HIV outcomes in this population which may also explain the high ART retention in the FSW cohort. This is consistent with what has been observed in Malawi and other SSA countries where the use of peers in enhancing ART adherence has been key in achieving high ART retention [7, 9].

This study demonstrated the impact of systemic and regional disparities on healthcare access in Nigeria by revealing notable differences in the uptake of ART among FSWs according to their state of residence. Compared to FSWs from other states, those from Niger State had a significantly higher chance of staying in care, which may be due to regional variables like better healthcare infrastructure, greater community support, or more successful Key Population Program implementation. These results are consistent with trends seen throughout SSA, where disparities in stigma levels, healthcare worker training, and resource allocation frequently cause ART adoption and retention to differ by region [49–51]. For instance, research conducted in SSA has highlighted the importance of community-based healthcare and decentralisation in enhancing ART access and adherence [52–54]. In order to guarantee universal access to HIV care for important groups throughout Nigeria and SSA, our findings highlight the significance of resolving state-level inequities through customised interventions, fair resource distribution, and focused legislative measures.

The results of this study highlighted the particular difficulties elder FSWs confront in sustaining regular access to antiretroviral medication, since they were more likely to be lost to follow-up than their younger colleagues, who were between the ages of 15 and 29. Our findings are consistent with other studies in similar settings where older ART patients were found to be more likely to be LTFU than their younger counterparts [55]. Additional obstacles that older FSWs may face include greater stigma, conflicting obligations to their families or jobs, or more serious health issues, all of which can make it difficult for them to adhere to their treatment plans [56]. These results are in line with data from Sub-Saharan Africa, where important groups have shown age-related differences in ART retention [57, 58]. For example, research conducted in Malawi and Zimbabwe has shown that mobile ART delivery strategies and peer-based support networks are more effective for younger people, and they might not adequately meet the needs of elderly populations [59]. This demonstrates the necessity of age-appropriate treatments to increase ART retention and health outcomes among older FSWs throughout SSA, including improved counselling, flexible clinic hours, and focused outreach. One strategy to increase retention amongst the older FSWs could be through use of reminders for medication re-fill and follow-up appointments [60]. Furthermore, engaging peer supporters or social workers to provide treatment continuation encouragement may also help reduce ART loss-to-follow-up amongst the older FSWs.

According to our current study, FSWs who moved from one state to another had a lower chance of being lost to follow-up than those who stayed in their current state of residency. This may have been due to the higher motivation for good health among migrating FSWs [61]. The migrating FSWs may have been prioritizing their health to ensure that they continued to work effectively, leading to proactively seek ART services which reduces the likelihood of being lost to follow-up. According to this research, FSW mobility may be associated with improved access to services, either as a result of proactive self-care practices among mobile populations or the availability of coordinated, migrant-friendly healthcare programs [62]. Studies from SSA have shown similar trends, with migration frequently linked to higher healthcare-seeking behaviours among critical populations, particularly when cross-regional programs serve mobile individuals [63]. To address the particular needs of migrants, for example, a South African study highlighted the significance of developing cross-border ART services [64]. In order to increase ART retention for mobile FSWs throughout Nigeria and SSA, these findings highlight the need for improved inter-state collaboration and continuity of care through decentralised and flexible healthcare systems.

Higher educated female sex workers had a lower likelihood of becoming LTFU in our study, underscoring the importance of education in enhancing treatment adherence, health-seeking behaviours, and overall retention in care. Higher educated people are better able to comprehend treatment plans, navigate healthcare systems, and overcome stigma, which improves ART retention, according to studies done throughout SSA [65]. The current study showed that LTFU was lower amongst the FSWs with formal education than those without formal education, which is consistent with a study conducted amongst FSWs in Somalia [65] where LTFU was lower amongst those with higher education. Another study conducted in Malawi also demonstrated lower LTFU amongst those with lower education [66]. In 2018, Ibiloye et al. reported that FSWs that had no formal education were 80% more likely to be LTFU compared to those with formal education [23]. Although level of education is associated with several inequalities in health [67], most of the studies that have investigated LTFU do not capture such information in Nigeria [68, 69] and other SSA countries [70, 71]. These findings underscore the importance of integrating educational empowerment initiatives into HIV care strategies to enhance ART retention and health outcomes across SSA.

The FSWs having hypertension were found to have higher LTFU compared to the FSWs without hypertension. This is one of the few studies to look at the effect of hypertension on ART LTFU amongst FSWs. There is evidence that regular hypertension treatment follow-up visits increase the likelihood of controlled blood pressure in patients with hypertension [72]. Therefore, this calls for the need of integration of non-communicable disease programmes, like hypertension screening and treatment, into the FSW HIV care cascade in order to improve the health more in general.

### Strengths and limitation of study

The large sample size and the study’s integration into routine service delivery enhance the reliability and programmatic relevance of the findings, offering useful insights for improving ART retention strategies and informing evidence-based policy decisions [73]. By leveraging real-world data from the Nigerian key population programme, the study provides practical evidence that can help guide interventions to reduce loss to follow-up, improve treatment adherence, and strengthen HIV care programmes for female sex workers.

However, several limitations should be considered when interpreting the findings. First, the study relied on routinely collected programme data, which may be subject to missing, incomplete, or inaccurately recorded information for some variables, despite the use of multiple imputation for selected covariates [74]. Second, some individuals classified as lost to follow-up may in fact have transferred to other health facilities without documentation, which could have resulted in outcome misclassification and an overestimation of LTFU [75–77]. Third, the study was conducted in only five states supported by the key population programme, and these may differ from other parts of Nigeria in terms of service delivery models, programme intensity, health system capacity, and characteristics of female sex workers. Therefore, the findings may not be generalizable to all female sex workers in Nigeria, particularly those in regions not covered by the key population programme. These limitations underscore the need for broader, nationally representative studies to capture regional variation and provide more comprehensive evidence for national policy and programming.

## Conclusion

In conclusion, female sex workers in Nigeria had a high retention on ART. Female sex workers aged ≥ 30 years, students, those with hypertension, married women, and those with no formal education had a higher likelihood of LTFU, whereas migrants, unemployed FSWs, those from Niger State, and those with normal BMI had a lower likelihood of LTFU. Therefore, Nigerian HIV programme should consider investigating the reasons for the high loss to follow-up especially in these FSW subgroups. This could be achieved through establishment of a robust system of patient tracking like the Back-to-Care (B2C) Programme [77]. Similar programmes in SSA may consider replicating the implementation modalities of ART retention amongst the Nigerian FSW programme in order to achieve optimal results.

## Data Availability

The dataset analysed during the current study are available from the corresponding author upon reasonable request.

## Abbreviations

AIDS: Acquired Immunodeficiency Syndrome
aSHR: Adjusted Subdistribution Hazard Ratio
ART: Antiretroviral Therapy
BMI: Body Mass Index
B2C: Back-to-Care
CBO: Community-Based Organization
CI: Confidence Interval
CIF: Cumulative Incidence Function
FCT: Federal Capital Territory
FG: Fine and Gray
FSW: Female Sex Worker
FSWs: Female Sex Workers
HALG: Heartland Alliance LTD/GTE
HIV: Human Immunodeficiency Virus
HIVST: HIV Self-Testing
IQR: Interquartile Range
KP: Key Population
KPs: Key Populations
LTFU: Loss to Follow-Up
MPPI: Minimum Prevention Package Intervention
MSM: Men Who Have Sex with Men
NACA: National Agency for the Control of AIDS
PLHIV: People Living with HIV
PEPFAR: President’s Emergency Plan for AIDS Relief
PrEP: Pre-Exposure Prophylaxis
PWID: People Who Inject Drugs
SSA: Sub-Saharan Africa
UNAIDS: Joint United Nations Programme on HIV/AIDS
VL: Viral Load
WHO: World Health Organization
